# Evaluating the impact of feeding dried distillers grains with solubles on Boer goat growth performance, meat color stability, and antioxidant capacity

**DOI:** 10.1093/tas/txac060

**Published:** 2022-05-09

**Authors:** Payton L Dahmer, Faith B McDonald, Colin K Y Chun, Charles A Zumbaugh, Cassandra K Jones, Alison R Crane, Tamra Kott, James M Lattimer, Michael D Chao

**Affiliations:** Kansas State University Department of Animal Sciences & Industry, Manhattan, KS 66506, USA; Kansas State University Department of Animal Sciences & Industry, Manhattan, KS 66506, USA; Kansas State University Department of Animal Sciences & Industry, Manhattan, KS 66506, USA; Kansas State University Department of Animal Sciences & Industry, Manhattan, KS 66506, USA; Kansas State University Department of Animal Sciences & Industry, Manhattan, KS 66506, USA; Kansas State University Department of Animal Sciences & Industry, Manhattan, KS 66506, USA; Kansas State University Department of Animal Sciences & Industry, Manhattan, KS 66506, USA; Kansas State University Department of Animal Sciences & Industry, Manhattan, KS 66506, USA; Kansas State University Department of Animal Sciences & Industry, Manhattan, KS 66506, USA

**Keywords:** antioxidant, dried distillers grains with solubles, goat, growth, meat color

## Abstract

A total of 72 male Boer goat kids (21.7 ± 0.5 kg) were fed for 21 d with 3 kids per pen and 12 pens per treatment. Dietary treatments were: 0% inclusion of dried distillers grains with solubles (DDGS; 0% DDGS) or 33% DDGS inclusion (33% DDGS) and were provided ad libitum. Goats and feeders were weighed weekly to collect body weights (BW) and determine feed disappearance in order to calculate average daily gain (ADG), average daily feed intake (ADFI), and feed efficiency (G:F). At the conclusion of the feeding study, a subset (*n* = 30; 2–3 goats from each pen representing six6 pens per treatment) of goats were harvested, carcasses evaluated, and loins were fabricated into 2.54 cm chops. Goat chop discoloration was evaluated by trained panelists and measured for L*, a*, and b* values on days 0, 4, 7, and 10 under retail display conditions. Samples were collected and analyzed for lipid oxidation, fatty acid profile, and hydrophilic and lipophilic antioxidant capacity. No evidence of differences was observed for final BW, ADFI, G:F, and carcass characteristics (*P* > 0.05). However, goats fed the 0% DDGS diet had greater ADG compared with those fed a diet containing 33% DDGS (*P* = 0.05). Overall, visual evaluation of discoloration, L*, a*, and b* as well as lipid oxidation data confirmed that feeding 33% DDGS to goats had no effect on goat chop discoloration and lipid oxidation (*P* > 0.10). However, all chops demonstrated a display effect, which they increased in visual discoloration and lipid oxidation and decreased in a* and b* values (*P* < 0.01) over the entirety of the 10-d period of retail display, regardless of the dietary treatments. As expected, feeding 33% DDGS to goats decreased relative percentage of multiple and total monounsaturated fatty acids, but increased relative percentage of multiple and total polyunsaturated fatty acids (PUFA; *P* < 0.05). The antioxidant capacity measurements showed no treatment difference in the hydrophilic portion (*P* > 0.10), but chops from the 33% DDGS treatment had greater lipophilic antioxidant activity compared with the 0% DDGS chops (*P* < 0.05). In conclusion, including 33% DDGS to the diet may negatively impact goat growth performance, but did not impact any carcass characteristics. Feeding a diet with 33% DDGS resulted in an increase in the PUFA content of goat chops but did not appear to impact meat color or lipid oxidation. The supposed negative consequence from increased PUFA is likely counterbalanced by the increased antioxidant capacity in the lipid component of meat, resulting in no difference in meat shelf-life.

## INTRODUCTION

The U.S. goat population has grown exponentially in recent years (USDA, 2010, 2020), along with an increase in ethnic populations and niche markets demanding goat meat (Spencer, 2008). To increase goat production through larger feeding operations, there is a need to explore economically viable feedstuffs for producers to formulate least-cost rations. Dried distillers grains with solubles (DDGS) are a byproduct of U.S. ethanol production that are commonly used in livestock feeding systems (Walter et al., 2012). Aside from its attractive pricing compared with corn or soybean meal, DDGS provide ample energy and rumen undegradable protein, making the feedstuff potentially suitable for goat diets (Schingoethe et al., 2009). However, the goat industry has yet to capitalize the use of DDGS as a feedstuff due to lack of published research surrounding its efficacy. In fact, the 2007 Nutrient Requirements of Small Ruminants lacks any information regarding feeding corn co-products, such as DDGS, to goats (NRC, 2007).

In addition to live performance, how DDGS can alter goat meat quality is also of great interest to goat producers, the corn industry, and the scientific community. Some past research has suggested that feeding high concentration of DDGS to beef cattle can increase the concentration of polyunsaturated fatty acids (PUFA) in beef, making the meat more prone to lipid oxidation and discoloration (Depenbusch et al., 2009; de Mello et al., 2012). Conversely, other studies have refuted these findings, but did not provide justification for the lack of adverse impact on beef shelf-life with an increase in PUFA (Roeber et al., 2005; Nade et al., 2012).

Work by Luthria et al. (2012) reported that the antioxidant capacity of DDGS was increased threefold compared with that of corn due to the concentration of lipophilic antioxidant from the removal of carbohydrate during the manufacturing process. The same authors also found high concentrations of phenolic acids such as caffeic, ferulic, p-coumaric, and sinapic acids, which are powerful antioxidants in DDGS. Supportive information was also provided by Hu et al. (2020) where the authors found DDGS supplementation to chickens can enhance antioxidant status in chicken plasma. Thus, if this increased antioxidant concentration in DDGS can be transferred to the meat of animals which consume DDGS, this would explain why feeding DDGS does not always translate to shortened shelf-life in meat, as the increased antioxidant in meat could mitigate the negative effects of increased PUFA. Unfortunately, there has been no published work in this area. Therefore, the objective of this study was to evaluate the efficacy of feeding DDGS to Boer goats and investigate any potential the impacts of feeding DDGS on goat meat shelf-life and antioxidant capacity.

## MATERIALS AND METHODS

All experimental procedures adhered to guidelines for the ethical and humane use of animals for research according to the Guide for the Care and Use of Agricultural Animals in Research and Teaching FASS (2012) and were approved by the Institutional Animal Care and Use Committee at Kansas State University (IACUC #4040.2).

### Animals, Diets, and Experimental Design

A total of 72 male Boer goat kids (21.7 ± 0.5 kg) were fed for 21 d to evaluate the impacts of feeding a diet with 33% DDGS on goat growth performance. Animals were housed in total confinement (3 m × 1.5 m pens) in an environmentally controlled (13 °C) building at the Kansas State University Sheep and Goat Center. On day 0 goats were individually weighed and allotted to pens (3 goats/pen and 12 replicate pens/treatment) in a completely randomized design. Each pen was randomly assigned to one of two dietary treatments: (1) a diet with no DDGS inclusion (0% DDGS) and (2) a diet with 33% inclusion of DDGS (33% DDGS). Diets (Table 1) were formulated to meet or exceed NRC (2007) nutrient requirements for 25 kg Boer doelings and male castrates gaining 100 to 150 g/h/d. Diets were pelleted with pellet binder included to combat any pellet quality issues. Alfalfa was included in the pellet as a form of roughage so that diets could be fed as a sole source ration. Each pen was equipped with a self-feeder and bucket waterer to provide ad libitum access to feed and clean water. Goats were fed experimental diets for 21 d with individual feeder weights and full goat weights collected weekly to determine average daily gain (ADG), average daily feed intake (ADFI), and feed efficiency (G:F). All feed additions were recorded daily. At the end of the feeding trial, 30 out of the 72 goats were selected for harvest at the KSU meat laboratory in Manhattan, Kansas. The harvest selection criteria were based on treatments and pen, resulting in 15 goat carcasses per treatment from six pens (2–3 goats per pen).

**Table 1.** Diet formulation and calculated nutrient composition (as-fed basis) of diets fed to Boer goats

	Dietary treament^1^	
Ingredient, %	0 % DDGS	33% DDGS
Corn DDGS	—	33.0
Soybean meal, 48% crude protein	11.94	—
Corn	15.00	2.40
Wheat middlings	26.40	8.30
Soybean hulls	39.00	48.64
Dehydrated alfalfa	3.00	3.00
Ammonium chloride	0.50	0.50
Limestone	1.97	1.97
Sodium chloride	1.00	1.00
Selenium selenite	0.01	0.01
Soybean oil	0.50	0.50
Vitamin A 30,000 IU	0.04	0.04
Vitamin D 30,000 IU	0.01	0.01
Vitamin E 20,000 IU	0.15	0.15
Trace mineral premix^2^	0.48	0.48
Total	100.00	100.00
Calculated analysis (as-fed basis)		
Crude protein, %	16.00	16.00
Crude fat, %	2.48	2.79
ADF, %	23.40	29.88
Net energy, Mcal/kg	2.23	2.23
Ca, %	1.05	1.00
P, %	0.40	0.40

Dietary treatments included either 0 or 33% dried distillers grains with solubles (DDGS).

Trace mineral premix provided: 0.30 mg/kg iron sulfate, 0.25 mg/kg zinc sulfate, 0.04 mg/kg cobalt, and 0.005 mg/kg manganese.

ADF = acid detergent fiber.

### Harvest and Fabrication

All 30 goats were harvested on the same day at the KSU meat laboratory by the meat laboratory manager and experienced student employees. The live weight was recorded prior to the harvest, and hot carcass weight (HCW) was immediately obtained after the harvest. Dressing % was calculated as


$$\begin{array}{l} Dressing\;\%=\frac{hot\;carcass\;weight}{live\;weight}\;\times 100 \end{array}$$

After 24 h of postmortem chilling at 2 ± 2 °°C, each carcass was ribbed between the 12th and 13th rib, and the following carcass characteristics were recorded: rib eye area (REA; cm^2^), body wall thickness (BWT; cm; measured at the 12th rib), and backfat thickness (BF; cm; measured at the 12th rib). Finally, both sides of the boneless loins (Longissimus lumborum) from all 30 carcasses were collected, vacuumed-packaged, and frozen at −40 °C until fabrication.

The loins were thawed at 2 ± 2 °C for 24 h prior to fabrication. Each loin was fabricated into four 2.54 cm chops designated for 0, 4, 7, or 10 d of retail display from the anterior to the posterior end of the loin muscle. All chops were overwrapped in Styrofoam trays (Dynea-Pak, Ontario, Canada) with oxygen permeable polyvinyl chloride film (HIY-45 Gold Stretch Meat film, O_2_ transmission rate = 1,191 cm^3^/0.065 m^2^/24 h; Berry Global Inc., Evansville, IN) and subjected to retail display conditions in coffin-style cases (Model DMF8; Tyler Refrigeration Corporation, Niles, MI) at 2 ± 2 °C. The chops were exposed to continuous 1,000 to 1,800 lux warm white fluorescence lighting (Model F32T8, 32 W, Warm White 3,000 K; Philips Lighting Company, Somerset, NJ) throughout the designated display period. Due to the small REA of goat chops, the left and the right loins from the same carcass were treated as one sample, resulting in 2 chops per package to ensure there was enough measurable area for the evaluation of objective color and subjective discoloration scores during the 10-d retail display period.

### Meat Color

Subjective discoloration was evaluated following the procedure described by Bloomberg et al. (2011). Briefly, a five-person trained panel subjectively evaluated discoloration of the 2 chops as a percentage (0%–100%; 0% = no discoloration, 100 = completely discolored/brown) of total surface area on days 0, 4, 7, and 10 of retail display. Panelists were trained using a visual discoloration guide consisted of 10 beef steak images ranging from 0% to 100% discoloration exemplifying surface discoloration with increments of 10%. Panelists were instructed to perform the evaluation at the same period each day to minimize variation. In addition, samples were randomly relocated once daily to minimize any location effects. Objective color measurements were obtained for CIE (standard color space for International Commission on Illumination) L*(brightness), a* (redness), and b* (yellowness) values using a using a Hunter Lab MiniScan EZ spectrophotometer (Model 4500L, Illuminant D65, 2.54-cm aperture, 10° observer; Hunter Associates Laboratory Inc., Reston, VA). The spectrophotometer was calibrated daily using a white and a black ceramic tile provided by the manufacturer, and color measurements were obtained on days 0, 4, 7, and 10 of display by averaging four readings from different areas of the 2 chops’ lean surface through the polyvinyl chloride film (2 readings per chop). At the end of the allotted display period, subcutaneous fat, and connective tissue were removed from all samples, and only the longissimus lumborum were pulverized in liquid nitrogen with a blender (model 51BL32, Waring Commercial, Torring, CT, USA) and stored at –80 °C until analysis.

### Fat Content and Fatty Acid Analysis

Lipid was extracted from the 30 samples from day 0 of retail display following the procedure described by Folch et al. (1957). Briefly, 1.2 mL of 1:1 chloroform/methanol was added to 0.3 g of pulverized tissue, homogenized, and combined with 1.2 mL of 0.8% KCl. The tubes were centrifuged at 1,000 × *g* for 5 min, and the bottom layer of each sample was transferred to a pre-dried and weighed glass tube using a Pasteur pipette. The content was evaporated to complete dryness under nitrogen, and excess moisture was further removed using a vacuum dryer (CentriVap DNA Vacuum Concentrator, Labconco, Kansas City, MO) for 1 h with no heat applied. Finally, dried lipid weight was obtained, and chloroform was added to each tube to achieve a lipid stock concentration of 4 mg/mL for each sample.

One-half milliliters of lipid stock from each sample were transferred to a screw cap vial, and 50 nmol of pentadecanoic acid (15:0) was added to each vial to serve as the internal standard. The fatty acid methyl esters were prepared following the procedure described by Ichihara and Fukubayashi (2010). The chloroform solvent from the lipid stock was again evaporated completely under constant nitrogen purge. One milliliter of 3 M methanolic hydrochloric acid was added to each tube, vortexed and heated at 78 °C for 30 min. Subsequently, 2 mL of ultrapure water and 2 mL of hexane were added, centrifuged (1,000 × *g* for 5 min), and the top hexane layer was carefully pipetted into a glass tube and evaporated to dryness. The fatty acid methyl esters were re-dissolved in 100 μL of hexane and transferred to 100 μL spring bottom vial inserts and inserted into the gas chromatography (GC) vials.

The GC was performed by an Agilent 6890N system with Flame Ionization Detector (FID; Agilent, Santa Clara, CA) at the Kansas Lipidomics Research Center at KSU. The GC was fitted with a DB-23 capillary column (column length—60 m, internal diameter—250 µm, film thickness—0.25 µm; Agilent). Helium was used as the carrier gas at a flow rate of 1.5 mL/min. The oven temperature ramp conditions were as follows: the initial temperature of 150 °C was held for 1 min, increased at 25 °C/min to 175 °C. Then it was increased at 4 °C/min to 230 °C, and maintained for 9 min. The FID detector was operated at 260 °C, the hydrogen flow to the detector was 30 mL/min and the air flow was 400 mL/min. The fatty acids were identified by comparison of retention times of the compounds in the sample with retention times of known commercial standards (37 component FAME mix, Supelco, Bellefonte, PA). Fatty acids were processed via Agilent Chemstation software (Agilent) and expressed as percentage of total fatty acids.

### Lipid Oxidation

All samples from all four retail display periods were subjected to lipid oxidation measurements. Lipid oxidation was determined by the thiobarbituric acid reactive substance (TBARS) assay described by Ahn et al. (1998) with modifications. Prior to sample preparation, a set of malonaldehyde (MDA) standards was prepared using malondialdehyde bisdiethyl acetal to produce the following concentrations: 25, 12.5, 6.25, 3.125, 1.5625 μM MDA and a blank. Approximately 0.1 g of pulverized tissue was homogenized with 700 µL of thiobarbituric acid/trichloroacetic acid (TBA/TCA) solution (15% TCA and 20 mM TBA) and 50 µL of 3% butylated hydroxytoluene (BHT) solution for 45 s using a bead homogenizer (D2400 Homogenizer, Benchmark Scientific, Edison, NJ). Samples were centrifuged at 2,000 × *g* for 5 min before being filtered through two layers of cheese cloth to remove floating fat and connective tissue particles. Six hundred microliters of MDA standards and filtered samples were transferred into 13 × 100 mm glass tubes. Tubes were incubated in a 70 °C water bath (Versa-Bath; Fisher Scientific; Waltham, MA) for 30 min before being cooled in ice cold water bath for 5 min. Finally, 0.2 mL of standards and the supernatant were plated on a 96-well plate in duplicate and read at an absorbance of 532 nm on a spectrophotometer (BioTek Eon; BioTek Instruments Inc., Winooski, VT). The final MDA concentrations were calculated and expressed as mg MDA/kg of muscle tissue.

### Antioxidant Capacity Measured by Oxygen Radical Absorbance Capacity

Oxygen radical absorbance capacity preparation. All samples from all four retail display periods were subjected to antioxidant capacity measurements measured by oxygen radical absorbance capacity (ORAC) assay according to the method described by Wu et al. (2008) with modifications. Approximately 0.1 g of pulverized tissue were homogenized for 45 s in 1 mL hexane using a D2400 bead homogenizer (Benchmark Scientific). Each tube was gently vortexed for 1 h, upon which the tubes were centrifuged at 3,000 × *g* for 10 min. The hexane layer was transferred to a glass vial, evaporated to dryness under nitrogen, and redissolved in 750 μL of 7% randomly methylated b-cyclodextrin (RMCD) in 50% acetone/50% water and preserved for lipophilic ORAC. For the preparation of the hydrophilic samples, 1 mL 20% ethanol/80% water solution was added to the same bead tube upon the removal of the hexane layer. Samples were again homogenized for 45 s and centrifuged at 12,000 × *g* for 10 min. The supernatant was preserved for the hydrophilic ORAC. Both lipophilic and hydrophilic portions were stored at –80 °C until analysis.

ORAC assay. One hundred microliters of hydrophilic componentwere diluted 20 times with phosphate buffer (75 mM sodium phosphate, pH 7.4). One hundred fifty microliters of diluted fluorescein solution (1:500 in phosphate buffer) were added to each well of a black 96-well plate (655906, Greiner Bio-One, Kremsmünster, Austria). Twenty-five microliters of Trolox standards (0, 6.25, 12.5, 25, 50, 100 μM) and the diluted samples were added to the plate in triplicates. The plate was incubated at 37 °C for 30 min in a fluorescent microplate reader (Synergy HT, Biotek Instruments Inc.), and 25 μL of 2,2ʹ-Azobis(2-methylpropionamidine) dihydrochloride (AAPH; 153 mM) was added to each individual well at the end of the incubation period. The 96-well plate was measured at an excitation wavelength of 485 nm and an emission wavelength of 520 nm every 1 min at a sensitivity of 60 for 120 min via Gen 5 software (BioTek Instruments Inc.).

The lipophilic component was further diluted 1:1 with 7% RMCD (50% acetone/50% water) prior to analysis. The same procedure followed in the hydrophilic ORAC assay was utilized here with the exception that 7% RMCD was used as the diluent instead of the phosphate buffer.

Final values for both the hydrophilic and lipophilic ORAC assays were determined by calculations of area under the curve (AUC) and net AUC via the Gen 5 software (BioTek Instruments Inc.). AUC and net AUC were calculated as


$$\begin{array}{l} AUC\;=\;0.5\;+\;\left(R2/R1\right)\;+\;\left(R3/R1\right)\;+\;\left(R4/R1\right)\;+\cdots +\;0.5(Rn/R1) \end{array}$$

$$\begin{array}{l} Net\;AUC\;=\;AUC\;sample\;\;AUC\;blank \end{array}$$

where R1 is the first fluorescence reading at the beginning of the reaction and Rn is the last fluorescence reading at the end of the 120 min. The AUC is that of a single sample, standard or blank, while the Net AUC is the net area under the fluorescein decay curve, which representing the antioxidant capacity of that sample. The final hydrophilic and lipophilic ORAC values were calculated by using a linear regression model generated using the Trolox standards, and data are expressed as µmol of Trolox equivalents (TE) per gram of muscle tissue (µmol of TE/g meat).

### Statistical Analysis

All data were analyzed using the GLIMMIX procedure of SAS (version 9.4, SAS Institute, Cary, NC) with pen as the experimental unit. All growth, carcass characteristics data and loin fat content were analyzed as a completely randomized design. All meat quality data, except meat color, were analyzed as a split-plot design with dietary treatments as the whole-plot and retail display day as the split-plot. Animals within diet was considered the whole-plot error term, and display day by diet was considered the split plot error term. Finally, meat color data were analyzed as a split-plot repeated-measures design with dietary treatments as the whole-plot and retail display day as the repeated-measures. The Toeplitz covariance structure was selected based on the best fit model. For all analysis, Kenward-Roger method was used to estimate the degrees of freedom, and separation of means was conducted using LSMEANS procedure (least significant differences) at *P* < 0.05.

## RESULTS AND DISCUSSION

### Growth Performance and Carcass Characteristics

Growth performance and carcass data are displayed in Tables 2 and 3, respectively. No evidence of differences (*P* > 0.05) was observed for final BW, ADFI, and G:F. Dietary treatment impacted goat ADG, where goats fed the 0% DDGS diet had greater (*P* = 0.05) ADG compared to those fed a diet containing 33% DDGS. DDGS inclusion at varying levels in the diet of small ruminants on ADG have shown inconsistent results in the literature. Paine et al. (2018) demonstrated a linear increase in Boer goat ADG and G:F with increasing inclusion of DDGS from 0% to 30%. Likewise, Crane et al. (2018) observed a linear increase in ADG as the level of DDGS inclusion increased from 0% to 45% in ram lamb diets. However, Felix et al. (2012) found that including DDGS beyond 20% negatively impacted ADG of feedlot lambs. The noted negative impact on performance was explained by inadequate rumination due to a lack of effective fiber in the pelleted diet, as well as reduced DM and fat digestibility. These authors found that supplemental forage in the diet attenuated the lack of ruminal activity. Goats in the present study were fed a complete pellet that included roughage, so no supplemental forage was provided. Perhaps, small ruminants like sheep and goat needed greater fiber level in their diet when DDGS is supplemented to ensure adequate digestion. Unfortunately, digestibility was not measured in this study, so it is difficult to conclude whether the supplementation of DDGS impacted digestibility and subsequent performance in this study.

**Table 2.** Effects of feeding a diet containing either 0% or 33% dried distillers grains with solubles (DDGS) on Boer goat growth performance^1^

	Dietary treatment			
Item	0% DDGS	33% DDGS	SEM	*P*-value
BW, kg				
Day 0	21.8	21.7	0.51	0.91
Day 21	26.6	25.8	0.51	0.28
ADG, kg/d				
Days 0–21	0.23	0.20	0.01	0.05
ADFI, kg/d				
Days 0–21	0.73	0.73	0.06	0.59
G:F				
Days 0–21	0.32	0.29	0.02	0.25

A total of 72 male Boer goats (initially 21.7 ± 0.51 kg BW) were used in a 21-d feeding study.

BW = body weight; ADG = average daily gain; ADFI = average daily feed intake; G:F = gain-to-feed ratio.

**Table 3.** Effects of feeding a diet containing either 0% or 33% dried distillers grains with solubles (DDGS) on Boer goat carcass characteristics^1^

	Dietary treatment			
Item;	0% DDGS	33% DDGS	SEM	*P* -value
Hot carcass weight, kg	13.4	13.5	0.43	0.88
Dressing percentage, %	47.23	48.13	1.00	0.53
Rib eye area, cm2	15.13	14.73	0.59	0.64
Body wall thickness, cm	1.39	1.34	0.07	0.63
Loin fat content, %	2.96	2.96	0.19	0.99
12th rib fat depth, cm	0.11	0.10	0.01	0.33

A subset of 30 goats (15 replicates per dietary treatment) were harvested upon conclusion of the feeding study at the Kansas State University Meat Lab.

There was no differences (*P* > 0.05) in HCW, dressing %, REA, BWT, BF, and loin fat content as a result of the diet (Table 3). Others have reported no impact of DDGS inclusion on carcass parameters in sheep (Van Emon et al., 2013; Crane et al., 2017). Conversely, Huls et al. (2006) replaced soybean meal with DDGS in finishing lamb diets and observed an increase in BF thickness from lambs fed 23% DDGS, which was attributed to the increased energy content. Diets in the current study were formulated to be isocaloric, but the 33% DDGS diet was higher in fat due to the increased corn oil from the DDGS. However, we saw no evidence that goats fed DDGS had increased fat deposition as a result of this feeding practice. Similarly, Mello et al. (2012) fed five different levels of modified distillers grains plus solubles (0%, 10%, 20%, 30%, 40%, and 50%) to steers in a feedlot setting for 176 days, and they found no difference in loin fat content among the treatments.

Due to constraints of the research facility, the goats in the current experiment were only fed for 21 d, whereas in many previous studies, animals were fed for longer periods (Hu et al., 2006; Solaiman et al., 2007; Sorensen et al., 2021). Therefore, this limited amount of time on feed could explain the lack of differences observed for carcass characteristics. Finally, it is important to note that we often refer to other species for information surrounding DDGS inclusion due to a lack of data in goat research, but it is critical to remember the species differences in lipid digestion and metabolism. It is known that when compared to other ruminants, goats tend to deposit far less subcutaneous, inter, and intra muscular fat (Adeyemi et al., 2015) and goats are not often fed to the same physiological age as beef cattle or sheep to reach the similar level of endpoint fat composition.

Fatty acid analysis. The fatty acid data are shown in Table 4. No evidence of difference (*P* > 0.05) in total saturated fatty acid (SFA) concentration was found between the 0% and 33% DDGS treatments.

**Table 4.** Fatty acid composition of loin chops (Longissimus lumborum) from goats fed a diet containing 0% or 33% dried distillers grains with solubles (DDGS)^1^

Item	0% DDGS	33% DDGS	SEM	*P-*value
Fatty acid, %				
14:0	1.67	1.31	0.12	<0.05
14:1	0.15	0.12	0.01	0.05
16:0	22.88	21.77	0.43	0.08
16:1	2.14	1.63	0.11	<0.01
17:0	1.04	0.91	0.04	<0.05
17:1	1.06	0.81	0.07	<0.05
18:0	13.20	14.32	0.44	0.09
18:1 *cis*	34.48	31.62	0.79	<0.05
18:1 *trans*	5.46	5.70	0.55	0.76
18:2	9.24	11.73	0.59	<0.01
18:3	0.78	0.91	0.05	0.07
20:1	0.12	0.11	0.005	0.45
20:3	0.39	0.50	0.03	<0.05
20:4	4.65	5.04	0.31	0.38
20:5	0.76	1.18	0.10	<0.01
22:0	0.07	0.07	0.007	0.40
22:4	0.36	0.29	0.02	<0.05
22:5	1.23	1.56	0.10	<0.05
22:6	0.31	0.41	0.05	0.14
Total SFA, %	38.87	38.39	0.62	0.59
Total MUFA, %	43.41	39.99	0.83	<0.01
Total PUFA, %	17.72	21.62	0.85	<0.01

Chops from goats fed a diet with 0% DDGS or 33% DDGS were analyzed for fatty acid composition at the Kansas Lipidomics Research Center at Kansas State University (Manhattan, KS).

Chops from goats fed 33% DDGS had lower concentrations of C14:0 and C17:0 compared with chops from goats fed a diet with 0% DDGS (*P* < 0.05). Feeding 33% DDGS to goats decreased the concentration of multiple monounsaturated fatty acids (MUFA), primarily on fatty acids C16:1, C17:1, and C18:1 *cis* and total MUFA (*P* < 0.05), but did not alter the concentration of C18:1 *trans* isomer (*P* > 0.05). At the same time, dietary intake of 33% DDGS increased the concentration of multiple polyunsaturated fatty acids, primarily on fatty acids C18:2, C20:5, and C22:5, and total PUFA (*P* < 0.05), but did not appear to change the concentration of C20:4 (*P* > 0.05).

The removal of the starch fraction of corn during distillers grains production can increase the protein and lipid content of DDGS to be three times more concentrated compared with corn (Srichuwong and Jane, 2011). Thus, DDGS can significantly alter the fatty acid composition of feeds due to the increase in corn oil concentration (Li et al., 2012; Wang et al., 2012; He et al., 2015). The primary fatty acid found in DDGS is linoleic acid (C18:2) (Ruan et al., 2018), and previous literature has concluded feeding a diet containing high levels of DDGS can alter the fatty acid profile in meat from various livestock species, with particular focus on the increase of C18:2 (Brandt et al., 1992; Gill et al., 2008; de Mello et al., 2012). This increase in C18:2 was also the main driver for the increased in total PUFA content for both the current and many other studies (Cromwell et al., 2011; Jiang et al., 2014).

Although microbes in the rumen tend to biohydrogenate unsaturated fatty acids (UFA) from the diet into SFA in ruminants, it is known that a portion of UFA bypass the rumen and can be directly deposited into tissues (Dugan et al., 2018). In addition, many studies have shown that passage rate and residence time of fatty acids in the rumen can impact the rate of biohydrogenation (Bauchart, 1993; Jenkins et al., 2008). Specifically, in fistulated cows, Beam et al. (2000) reported that C18:2 was biohydrogenated at a rate of 14.3%/h, but this rate significantly declined as the inclusion of C18:2 increased.

The C18:1 *trans* is generated from the biohydrogenation of PUFA by microorganisms in the rumen (Smith et al., 2009). The lack of difference in C18:1 *trans* between chops from goats fed 0% and 33% DDGS in the current study indicated that the rate of biohydrogenation likely did not play a role in the alteration of fatty acid composition of the meat. The PUFA in DDGS have been shown to escape ruminal biohydrogenation, which results in an increase in duodenal UFA and a subsequent increase in their efficiency of utilization (Vander Pol et al., 2009; Xu et al., 2014). This increase in PUFA by-pass from the rumen could explain the increase in C18:2 that the current study observed in chops from goats fed 33% DDGS.

Meat color. Overall, all chops demonstrated a display effect (*P* < 0.01), which they increased in visual discoloration and decreased in both a* and b* values over the entirety of the 10-d period of retail display, regardless of the dietary treatment (Table 5). Discoloration of chops was minimal early in subjective evaluation (*P* > 0.05; 0 and 0.27% discoloration on days 0 and 4, respectively). However, chops were more discolored by day 7 (*P* < 0.05; 11.94% discolored on day 7) and this trend continued through day 10 of retail display (55.55%). Furthermore, visual evaluation of discoloration and meat color characteristics measured by L*, a*, and b* values confirmed that no evidence of a dietary treatment effect (*P* > 0.05) was observed for goat chop discoloration (Table 6).

**Table 5.** Effect of retail display length on goat loin chops (Longissimus lumborum) discoloration, lipid oxidation, and antioxidant capacity^1^

	Display day^2^					
Item	0	4	7	10	SEM	*P*-value
Discoloration %^3^	0.00^c^	0.27^c^	11.94^b^	55.55^a^	2.08	<0.01
L*	35.19	33.97	34.87	34.31	0.75	0.55
a*	10.70^a^	8.02^b^	6.36^c^	5.64^d^	0.22	<0.01
b*	11.61^a^	11.25^a^	11.10^a^	10.38^b^	0.31	<0.01
Lipid oxidation, mg MDA/kg meat	0.45^c^	0.51^bc^	0.90^b^	1.71^a^	0.15	<0.01
Hydrophilic ORAC, µM TE/g of meat	10.32^d^	13.31^c^	14.65^b^	16.15^a^	0.39	<0.01
Lipophilic ORAC, µM TE/g of meat	0.65^c^	0.75^bc^	0.77^b^	1.05^a^	0.04	<0.01

Means that do not share a common superscript differ, *P *< 0.05.

Chops from 30 goats were displayed for 10 d and evaluated for subjective and objective color measurements, lipid oxidation, and hydrophilic and lipophilic antioxidant capacity.

At fabrication, chops were assigned to one of four retail display lengths: 0, 4, 7, or 10 d of retail display.

Subjective discoloration was evaluated as described by Bloomberg et al. (2011). Briefly, a five-person trained panel subjectively evaluated discoloration of the 2 chops as a percentage (0%–100%; 0% = no discoloration, 100 = completely discolored/brown).

**Table 6.** Effect of dietary treatment on goat loin chops (Longissimus lumborum) discoloration, lipid oxidation, and antioxidant capacity^1^

	Dietary treatment^2^			
Item	0% DDGS	33% DDGS	SEM	*P*-value
Discoloration %^3^	17.79	16.09	1.28	0.36
L*	34.51	34.66	0.77	0.89
a*	7.81	7.54	0.18	0.30
b*	11.19	10.96	0.35	0.66
Lipid oxidation, mg MDA/kg meat	0.85	0.94	0.11	0.57
Hydrophilic ORAC, µM TE/g of meat	13.53	13.68	0.27	0.71
Lipophilic ORAC, µM TE/g of meat	0.76	0.85	0.03	<0.05

Chops from 30 goats were evaluated for subjective and objective color measurements, lipid oxidation, and antioxidant capacity.

Goats were fed a diet with either 0% or 33% inclusion of dried distillers grains with solubles (DDGS).

Subjective discoloration was evaluated as described by Bloomberg et al. (2011). Briefly, a five-person trained panel subjectively evaluated discoloration of the 2 chops as a percentage (0%–100%; 0% = no discoloration, 100 = completely discolored/brown).

This result was expected, as it is well understood that prolonged display time in combination with exposure to both oxygen and light can hinder color stability of meat (Seideman et al., 1984). Specifically, pro-oxidants such as the oxygen and light can stimulate the oxidation of oxymyoglobin, which involves the loss of an electron of the iron molecule and transform it from ferrous to ferric state. This will transform the bright cherry red oxymyoglobin into the brownish color associated with metmyoglobin, resulting in the discoloration of meat (Seideman et al., 1984). Many studies have documented decreases in meat color stability during retail display when the animals were supplemented with DDGS or other corn co-products (Cortinas et al., 2005; Koger et al., 2010; Schilling et al., 2010). However, we did not see evidence of a dietary treatment effect on goat chop discoloration. It is important to point out many other studies have also reported findings similar to ours, stating that various types of DDGS supplementation have no effect on meat shelf-life (Soares et al., 2012; Domenech-Pérez et al., 2017; Chao et al., 2018), but these papers failed to provide a meaningful explanation for the lack of discoloration among various dietary treatments. Song et al. (2013) noted a marked decrease in oxidative stress in pigs fed DDGS and commented that this improvement in antioxidant capacity may have been driven by the increased sulfur concentration of the DDGS, whereby the sulfur could stimulate the synthesis of sulfur-containing antioxidant peptides.

Lipid oxidation. As expected, a display effect (*P* < 0.01) was found for lipid oxidation, where the MDA concentration increased as the display days increased (Table 5). Lipid oxidation followed the same trend as discoloration, where no evidence of a dietary treatment effect was found (*P* > 0.05; Table 6) between chops from goats fed 33% DDGS and 0% DDGS. This positive relationship between display time for meat products and lipid oxidation is well established in many past studies (Mohamed et al., 2008; Johnson and Decker, 2015; Ponnampalam et al., 2017). In a review on lipid oxidation, Domínguez et al. (2019) pointed out that both oxygen and light from the retail display can stimulate the production of peroxy-radicals, which can take hydrogen ions from the fatty acids in meat, particularly at the location where it is next to a double bond in a carbon chain. This begins a free-radical chain reaction that eventually leads to the increase production of secondary lipid oxidation product, such as MDA, as seen in the current study.

Previous studies also showed that an increase in PUFA concentration will also increase the subsequent lipid oxidation in meat (Cortinas et al., 2005; de Mello et al., 2012; Domínguez et al., 2019). However, other studies failed to find differences in lipid oxidation for samples with distinct fatty acid profiles as seen in this current study (Roeber et al., 2005; Nade et al., 2012). Most of the time, the noted inhibition of lipid oxidation during retail display in meat is due to some level of antioxidant supplementation, with the most notable dietary antioxidant being α-tocopherol for many different livestock species (Yang et al., 2002; Insani et al., 2008; Boler et al., 2009). However, what is often neglected is the inherent antioxidant level in the feed ingredients themselves. For example, corn is inherently rich in many phenolic acid-type antioxidants such as caffeic and ferulic acids (Luthria et al., 2012; Shin et al., 2018). During the processing of distillers grains, many nutritional components of corn, such as protein and fat, become highly concentrated in the resulting byproduct (Martinez-Amezcua et al., 2007), and research has found DDGS to have higher antioxidant capacity than ground corn (Shin et al., 2018). It is possible that the native antioxidants in corn can also be concentrated during the distillers grains manufacturing process. Perhaps, the DDGS contain a higher concentration of antioxidant that may be transferred to the meat which can potentially counteract the negative impacts of the increase PUFA content in the goat chops.

Antioxidant capacity. To investigate the antioxidant capacity of goat chops from the different dietary treatments, the oxygen radical absorbance capacity (ORAC) was used. The ORAC assay, originally developed by Cao et al. (1993), is based upon the previously mentioned peroxyl-radical oxidative reaction, which accounts for both oxidation inhibition time and the degree of inhibition (Huang et al., 2002). Furthermore, Prior et al. (2003) showed that the ORAC method can be used to measure the antioxidant capacity from both the hydrophilic and lipophilic portions of the samples with small modifications steps, which is suitable for this study to determine the source/location of the incorporation of the antioxidants.

Display day impacted (*P* < 0.01) both the hydrophilic and lipophilic ORAC, where in both cases the ORAC increased (Table 5) from days 0 to 10 of retail display. Since this assay includes both inhibition time and the degree of inhibition, this response was expected and further validates the use of ORAC in this study. The ORAC displayed no evidence of a treatment effect in the hydrophilic portion (*P* > 0.05), but chops from the 33% DDGS dietary treatment had greater lipophilic antioxidant capacity compared with the chops from goats fed 0% DDGS (*P* < 0.05; Table 6). Antioxidants can be either water-soluble or fat-soluble (Papas, 1993). Lipophilic antioxidants, such as α-tocopherols, are incorporated into the phospholipid cell membrane, where they have shown to be a strong mitigant against lipid oxidation in meat (Descalzo and Sancho, 2008). While no specific antioxidant was supplemented in our study, our data indicated a stronger presence of lipophilic antioxidant capacity in the goat chops from animals fed 33% DDGS. Other studies such as Wu et al. (2008) reported an increase in lipophilic antioxidant status in beef steers finished on a forage-based diet compared with concentrate, while Hu et al. (2020) fed chickens either a corn or DDGS-based diet, where the lipophilic ORAC of the chicken plasma was higher from animals fed DDGS.

The higher lipophilic ORAC would explain the lack of difference in MDA concentration between chops from 0% DDGS and 33% DDGS-fed goats, even with the notable increase in PUFA content. However, findings from these types of studies are rather inconsistent as there is considerable variation in DDGS antioxidant capacity from batch to batch due to heating differences during the manufacturing process (Shin et al., 2018). This could explain the inconsistency among published data investigating the effect of distillers grains inclusion on meat quality from various livestock species, as discussed in the introduction.

## CONCLUSIONS

In summary, including 33% DDGS to the diet negatively impacted goat growth performance, but did not impact any carcass characteristics. Feeding a diet with 33% DDGS resulted in an increase in the PUFA content of goat chops but did not appear to impact meat color or lipid oxidation. Our data suggest that this was due to an increase in the lipophilic antioxidant capacity of chops from goats fed the 33% DDGS, which likely counterbalanced negative effect from the increased PUFA content in meat. With an extremely limited supply of published literature focusing on the antioxidant capacity of distillers grains, further research is necessary to reevaluate the effect distillers grains supplementation has on meat quality.
